# Sesamin Exerts an Antioxidative Effect by Activating the Nrf2 Transcription Factor in the Glial Cells of the Central Nervous System in *Drosophila* Larvae

**DOI:** 10.3390/antiox13070787

**Published:** 2024-06-28

**Authors:** Akihiro Tsuji, Eiji Kotani, Yoshihiro H. Inoue

**Affiliations:** Biomedical Research Center, Graduate School of Science and Technology, Kyoto Institute of Technology, Matsugasaki, Sakyo, Kyoto 606-0962, Japan; m2641020@edu.kit.ac.jp (A.T.); kotani@kit.ac.jp (E.K.)

**Keywords:** sesamin, Nrf2, glial cells, cytochrome P450, *Drosophila*

## Abstract

Sesame seeds are abundant in sesamin, which exerts health-promoting effects such as extending the lifespan of adult *Drosophila* and suppressing oxidative stress by activating the Nrf2 transcription factor. Here, we investigated whether sesamin activated Nrf2 in larval tissues and induced the expression of Nrf2 target genes. In the sesamin-fed larvae, Nrf2 was activated in the central nervous system (CNS), gut, and salivary glands. The ectopic expression of Keap1 in glial cells inhibited sesamin-induced Nrf2 activation in the whole CNS more than in the neurons, indicating that sesamin activates Nrf2 in glia efficiently. We labeled the astrocytes as well as cortex and surface glia with fluorescence to identify the glial cell types in which Nrf2 was activated; we observed their activation in both cell types. These data suggest that sesamin may stimulate the expression of antioxidative genes in glial cells. Among the 17 candidate Nrf2 targets, the mRNA levels of Cyp6a2 and Cyp6g1 in cytochrome P450 were elevated in the CNS, gut, and salivary glands of the sesamin-fed larvae. However, this elevation did not lead to resistance against imidacloprid, which is detoxified by these enzymes. Our results suggest that sesamin may exert similar health-promoting effects on the human CNS and digestive tissues.

## 1. Introduction

Living organisms are constantly exposed to oxidative stress that is generated both inside and outside the body. Excessive oxidative stress damages lipids, proteins, and DNA [1,2]. Furthermore, oxidative stress is closely linked to the pathogenesis of diseases such as cancer and diabetes [3]. Organisms possess antioxidant systems that produce antioxidant enzymes and antioxidants [4,5]. One of the most critical antioxidant systems is the Keap1–Nrf2 system for transcriptional regulation. The transcription factor Nf-E2-related factor (Nrf2) plays an indispensable role in maintaining redox homeostasis in the body. Its target genes include those encoding enzymes involved in oxidative stress removal, detoxification, metabolism, and other crucial metabolic pathways [6]. Under non-oxidative stress, Nrf2 forms a complex with the repressor Kelch-like ECH-associated protein 1 (Keap1) and is immediately ubiquitinated and degraded by proteasomes. In contrast, under oxidative stress, the SH group of Keap1 is oxidized, eliminating the interaction between Nrf2 and Keap1. This process dissociates the inhibitor and stabilizes Nrf2, which migrates to the nucleus. There, it binds to a consensus sequence called the antioxidant response element (ARE), which promotes the transcription of target genes [7,8,9,10,11]. An experimental system was developed to monitor Nrf2/Cnc activation in the *Drosophila* body by creating an *ARE–GFP* reporter with a *GFP* gene downstream of this sequence [12]. Generally, Keap1–Nrf2 binding is disrupted by reactive oxygen species (ROS) generated during metabolic processes or by external environmental stimuli such as UV light or some chemicals. This disruption activates Nrf2, which promotes the transcription of antioxidant genes and protects the cells from oxidative stress [13,14,15,16,17]. Thus, the Keap1–Nrf2 system protects organisms against endogenous and exogenous oxidative stress. In mice, Nrf2 expression in the liver decreases with age [18]. Additionally, Nrf2 deficiency increases oxidative stress and promotes aging [19]. Therefore, identifying effective anti-oxidation and aging substances in the diet, as well as the active consumption of these substances, is crucial for preventing oxidative damage-associated diseases and delaying the progress of aging.

Among the lignans that are abundant in sesame seeds, sesamin has been particularly well studied for its potential as a supplement owing to its health-promoting effects [20,21,22,23]. A previous study demonstrated that sesamin prolonged the lifespan of adult *Drosophila* by inhibiting and delaying the aging phenotype of muscles, midgut stem cells, and neurons [24]. Additionally, it mitigated the age-dependent loss of dopaminergic neurons due to the accumulation of oxidative stress in the adult brain, suggesting that the antioxidant effect of sesamin is closely linked to its anti-aging effects [24,25]. Although several in vitro and in vivo studies have demonstrated the antioxidant effect of sesamin, direct evidence indicating its anti-aging effect in mammals, including humans, is yet to be obtained [26,27,28,29,30]. In contrast, studies on *Drosophila* conducted by our group have shown that the antioxidant effect of sesamin is mediated by Nrf2 activation [24,25]. Oxidative damage to intracellular biomaterials inflicted by endogenous reactive oxygen species has been proposed to be a factor in the aging process [31]. The results obtained for *Drosophila* adults provide important insights into the mechanism underlying the anti-oxidative and anti-aging effects exerted by sesamin. During metamorphosis, the central nervous system (CNS) of *Drosophila* undergoes a massive reorganization, changing from the larval neural network to the adult one. It is interesting to examine whether sesamin enhances Nrf2 activation in the CNS during the third instar larval to pupal stages, as cell proliferation and differentiation within the CNS and neural network reorganization are more active than in the adult stage. After hatching, *Drosophila* larvae grow from first instar larvae to third instar larvae, the terminal instar stage, over a period of about 5 days before pupation. When the larvae are fed a chemical mixed into their diet, this chemical’s effect on development can easily be studied [24,25]. In this study, we investigated whether sesamin activates Nrf2 using the *ARE–GFP* reporter that enables the monitoring of Nrf2 activation with high sensitivity [12].

Neurons and glial cells are heavily involved in age-related neurodegenerative diseases [32]. Although sesamin exerts a suppressive effect on the oxidative damage of the nervous system, few studies have examined its effects on glial cells in the CNS using mice and rats. *Drosophila* models are also being increasingly used because of their nervous system’s high similarity to that of mammals. Although the number of neurons in the *Drosophila* CNS is 1000–10,000 times less than that in mammals [33], glutamate, dopamine, serotonin, acetylcholine, GABA, and other neurotransmitters are used in the CNS for neural activity as they are in humans. Additionally, several genes and regulators involved in CNS development and function are shared between humans and *Drosophila* [34]. Drug metabolism, mechanisms of action, and the effects on neural activity and behavior in *Drosophila* are similar to those in mammals [35]. Therefore, using *Drosophila* provides a more convenient way to study the effects of sesamin on the developing CNS.

In addition to its anti-oxidative effect on the CNS of *Drosophila*, the consumption of sesamin suppresses the oxidative damage-dependent phenotype that appears in the gut of *Drosophila* adults [24]. Sesamin is also metabolized to its intermediate metabolite SC1 [24], which is absorbed and metabolized in intestinal epithelial cells in *Drosophila* adults, similar to what is observed in mammals. The *Drosophila* gut is responsible for the digestion and absorption of nutrients. It gradually becomes less functional with age owing to an activated inflammatory response and metabolic decline [36]. In contrast, the larval intestinal gut undergoes degradation during metamorphosis and reconstruction into the adult gut [37]. Investigating the effects of sesamin on Nrf2 activation in the larval intestinal epithelial cells helps us to understand the roles of this transcription factor on tissue reconstruction and to examine whether this chemical is effective in negating cellular stresses that occur during development.

Moreover, metabolic enzymes such as CYP450 are abundantly expressed in intestinal epithelial cells [38]. For instance, the oxidative stress-induced transcription of *Cyp2* in mammalian cells is initiated via the binding of Nrf2 to the ARE sequences in the 5’ untranslated region of this gene. Nrf2 depletion downregulates P450 gene expression [39,40,41], affecting the maintenance of a drug’s concentration in the blood. The *Cyp6g1* gene, a *Drosophila* orthologue of the mammalian *Cyp3A4* gene, is implicated in the metabolism and detoxification of dichlorodiphenyltrichloroethane (DDT) and imidacloprid, which have been used for many years as insecticides. Furthermore, in a DDT-resistant strain, the overexpression of the genes and/or raised activities of the enzymes were observed [42,43]. Therefore, if sesamin can activate the Nrf2, it is worth examining whether it may ultimately confer resistance to these insecticides through the induction of its target detoxification genes.

In this study, based on findings regarding the effects of sesamin on *Drosophila* adults, we investigated whether sesamin induces the activation of the transcription factor Nrf2 in larvae. We also investigated the subsequent antioxidant responses in the CNS and digestive tissues during larval development. Sesamin-fed larvae showed Nrf2 activation in the CNS, salivary glands, and gut. Next, we investigated whether the neurons or glial cells within the CNS of sesamin-fed larvae presented stronger Nrf2 activation. Consequently, we found that sesamin activated Nrf2 in glial cells rather than in neurons in the larval CNS. This study discusses how Nrf2 activation in glial cells influences CNS development. Next, we examined the endogenous genes whose transcription was altered by sesamin. We found that the mRNA levels of several drug-metabolizing enzymes were increased in the CNS, gut, and salivary glands of the sesamin-fed larvae. Since the upregulation of these enzymes has been reported in *Drosophila* mutant strains that are resistant to a neurotransmitter-blocking insecticide, we further investigated whether sesamin influences sensitivity to the drug in question. Based on the antioxidant and anti-aging effects observed in adults, it was surmised that sesamin mediates antioxidant and detoxifying effects via Nrf2 activation during larval development. These results suggest that sesamin may exert similar effects on the human CNS and digestive tissues. This is an important finding with regard to the health benefits of sesamin.

## 2. Materials and Methods

### 2.1. Fly Stocks and Culture

To monitor ARE-dependent transcription, the ARE-GFP reporter (ARE-GFP), in which the cDNA for GFP was placed after ARE sequences on which the Nrf2/Cnc transcription factor binds, was used. Stock harboring the *ARE-GFP* was given as a gift by D. Bohmann (University of Rochester Medical Center, Rochester, NY, USA). *P{EPgy2}Keap1[EY02632]* (*UAS-Keap1*) (#15427) was used for the overexpression of *Keap1*. The Gal4/UAS system was used for ectopic gene expression in *Drosophila*. The following fly stocks were obtained from the Bloomington *Drosophila* Stock Center (Bloomington, IN, USA). To visualize all neurons in larval CNSs, we used *P{GawB}elavC155*; *P{tubP-GAL80^ts^}10* (*elav-Gal4*) (#67058). To visualize specific types of glial cells in larval brains, the following Gal4-drivers were used: *P{GAL4}repo* (*repo-Gal4*) (#7415) for expression in all glial cells, *P{Eaat1-GAL4.R}2* (*Eaat1-Gal4*) (#8849) for expression in astrocytes, and *P{nrv2-GAL4.S}3. P{nrv2-GAL4.S}8* (*Nrv2-Gal4*) (#6797) was used for expression in the cortex and surface glial cells. The *P{UAS-tdTom.S}3* (*UAS-RFP*) (#36328) stock was used in combination with the appropriate Gal4 driver for labeling the neurons and the glial cells via the expression of Red Fluorescence Protein (RFP).

A standard cornmeal diet consisting of 7.2 g of agar, 100 g of glucose, 40 g of dried yeast, and 40 g of cornmeal per liter was used as fly food. All the ingredients were mixed well and boiled for 10 min, as previously described [44]. After cooling to 75 °C, 5 mL of 10% parahydroxybenzonate dissolved in ethanol and 5 mL of propionic acid were added as anti-septic agents to the diet; all fly stocks were maintained on a regular cornmeal diet at 25 °C, except for during the overexpression experiments, which were performed at 28 °C.

### 2.2. Chemical Feeding

For sesamin feeding, early third instar larvae were collected and separated into males and females. The larvae were reared in a single plastic vial containing *Drosophila* instant medium (Formula 4–24^®^ Instant *Drosophila* Medium, Blue; Carolina Biological Supply Company, Burlington, NC, USA) at a 0.3 g/mL concentration. Sesamin (Nacalai Tesque, Kyoto, Japan) dissolved in DMSO solution (Dimethyl sulfoxide; Wako Pure Chemical Industries, Ltd., Osaka, Japan) was added to final concentrations of 1 mg/mL of sesamin and 1% DMSO in the instant medium. As a control diet, 1% DMSO alone was added to the instant medium. For the imidacloprid feeding experiment, adults were mated while adhering to a standard cornmeal diet supplemented with 1 mg/mL sesamin and 1% DMSO or the diet with 1% DMSO alone as a control, and was then removed after 8 h of egg laying. A total of 72 h later, larvae were collected and reared on a standard cornmeal diet containing 3 µM of imidacloprid (Tokyo chemical industry, Tokyo, Japan) and 1 mg/mL of sesamin with 1% DMSO. The individuals were kept at 25 °C.

### 2.3. Observation of GFP and RFP Fluorescence

To visualize GFP fluorescence in the whole body, the *ARE-GFP* larvae at the early third instar stage were collected and fed the instant medium supplemented with sesamin (1 mg/mL) and DMSO (1%) for 24 h, while the medium with 1% DMSO alone was used as a control diet. GFP fluorescence was observed under a stereo fluorescence microscope (SZX7; Olympus, Tokyo, Japan). To visualize GFP and/or RFP fluorescence in the CNSs, guts, and salivary glands, third instar larvae harboring the *ARE-GFP* were collected and fed sesamin (1 mg/mL) and DMSO (1%) for 24 h. To visualize neurons or glial cells in the larval CNSs, the flies carrying pan-neuronal, neuron-specific, pan-glial, or glial-specific Gal4 drivers were crossed with those with UAS-RFP. Third instar larvae were collected and raised for 24 h on diets supplemented with 1 mg/mL of sesamin and 1% DMSO. The CNS, gut, and salivary glands were collected from the larvae, as previously described [24,44]. The whole CNSs, gut, or salivary glands were fixed in 4% paraformaldehyde for 30 min, washed with PBST (phosphate-buffered saline containing 0.1% Triton X-100), and subsequently blocked with 10% normal goat serum for 30 min. All the samples were mounted in Vectashield (Vector Laboratories, Burlingame, CA, USA). GFP and RFP fluorescence were observed using an Olympus laser scanning confocal microscope (Fv10i; Olympus, Tokyo, Japan) or a stereo fluorescence microscope (SZX7; OLYMPUS, Tokyo, Japan). The brightness and contrast of the entire images were adjusted using Fv10i software (Ver.03.01).

### 2.4. Survival Rate of Larvae Fed the Diet Supplemented with Drugs

After the adults had completed their 8 h egg-laying period, the hatched larvae were reared on a standard cornmeal diet supplemented with 1 mg/mL of sesamin and 1% DMSO. The late second instar larvae were collected and transferred to a standard cornmeal diet with imidacloprid and 1 mg/mL of sesamin with 1% DMSO. Then, the number of pupae was counted, and the survival rate from larvae to pupa was calculated.

### 2.5. Quantitative Reverse Transcription Polymerase Chain Reaction

For quantitative reverse transcription polymerase chain reaction (qRT-PCR) analysis, total RNA was extracted using TRIzol reagent (Invitrogen, Carlsbad, CA, USA) from the CNSs, gut, and salivary glands of larvae fed diets with or without 1 mg/mL of sesamin for 24 h. cDNA synthesis was performed using a PrimeScript II High Fidelity RT-PCR kit (Takara, Kusatsu, Japan) with oligo dT primers. qRT-PCR was performed using TB Green Premix Ex Taq II (Takara, Kusatsu, Japan) and the Thermal Cycler Dice^®^ Real Time System III (Takara, Kusatsu, Japan). RP49 was used as the normalization reference. The relative mRNA levels were quantified using the Thermal Cycler Dice^®^ Real Time System III version 6.0.1 (Takara, Kusatsu, Japan). The primers used were as follows:

RP49-Fw, 5′-TTCCTGGTGCACAACGTG-3′,
RP49-Rv, 5′-TCTCCTTGCGCTTCTTGG-3′,
GFP-Fw, 5′-AAGCTGACCCTGAAGTTCATCTGC-3′,
GFP-Rv, 5′-CTTGTAGTTGCCGTCGTCCTTGAA-3′,
Cyp6a2-Fw, 5′-TTCACCACCGATGTGATTGGC-3′,
Cyp6a2-Rv, 5′-TCGGGCATCATGCGCATT-3′,
Trx2-Fw, 5′-ATGGACAGCTGACCAAGGCATC-3′,
Trx2-Rv, 5′-CCCACTTAGATATTGGCCTTGATG-3′,
GPx-Fw, 5′-GGTCGATGTGAATGGAGACA,
GPx-Rv, 5′-CCCTCCTTGTTCACCAGAAA,
Sod3-Fw, 5′-AGCTGGAGGGATTGAAGGAG-3′,
Sod3-Rv, 5′-GGGGCCACCGTGATCAAC-3′,
Gclm-Fw, 5′-AGGATTCCAACGTCAGCAGG-3′,
Gclm-Rv, 5′-AATCTGCTGCTTGAGGGCAT-3′
ND-20-Fw, 5′-TTTCTCCTGGTGCCATTACC-3′,
ND-20-Rv, 5′-CTGCAGCAGGATAGGTCCTC-3′,
TrxR1-Fw, 5′-CGTTCTATTGTGCTGCGTGG-3′,
TrxR1-Rv, 5′-AGCTTGCCATCATCCTGCTT-3′,
Catalase-Fw, 5′-TTTCTCCTGGTGCCATTACC-3′,
Catalase-Rv, 5′-CTGCAGCAGGATAGGTCCTC-3′
gb-Fw, 5′-CCATGAGGGGTATGATCAGTG-3′,
gb-Rv, 5′-ATTTATGTGCTGGCCAATGTG-3′,
Aldh1-Fw, 5′-TCCGAGGGAGATAAGGCTGA-3′,
Aldh1-Rv, 5′-GAATGCCTTGTCCCGATCCA-3′,
Jafrac1-Fw, 5′-ACCGAGATCATTGCGTTCTC-3′,
Jafrac1-Rv, 5′-AAGTGGGTGAACTGGCTGTC-3′,
Gclc-Fw, 5′-ATGACGAGGAGAATGAGCTG-3′,
Gclc-Rv, 5′-CCATGGACTGCAAATAGCTG-3′,
Pgd-Fw, 5′-GGAATGTGTGAACGGGAAAGTGGAG-3′,
Pgd-Rv, 5′-AGGACTCGTGGCGCGAGGTG-3′,
Adh-Fw, 5′-AAACTGGCCCCCATTACCG-3′,
Adh-Rv, 5′-CAAGTCCAGTTTCCAGATG-3′,
HO-Fw, 5′-ACCATTTGCCCGCCGGGATG-3′,
HO-Rv, 5′-AGTGCGACGGCCAGCTTCCT-3′,
Zw-Fw, 5′-AAGCGCCGCAACTCTTTG-3′,
Zw-Rv, 5′-AGGGCGGTGTGATCTTCC-3′,
Ref(2)p-Fw, 5′-CGTAAGGACCTTCTGGATCG-3′,
Ref(2)p-Rv, 5′-CGTCGTGGATGGTGAAATTG-3′,
Cyp6g1-Fw, 5′-GCCCGCTGCGATCCCCAT-3′,
Cyp6g1-Rv, 5′-CCT TTCCAATCTCCTGCATA-3′,

The gene accession numbers for each gene were as follows: *RP49* (gene accession number: FLYBASE: FBgn0002626), *GFP* (GenBank: L29345.1), *Cyp6a2* (FBgn0000473), *Trx2* (FBgn0040070), *GPx* (FBgn0035438), *Sod3* (FBgn0033631), *Gclm* (FBgn0046114), *ND-20* (*NADH dehydrogenase (ubiquinone) 20 kDa subunit* (FBgn0030718), *TrxR1* (FBgn0020653), *Catalase* (FBgn0000261), *genderblind* (*gb*) (FBgn0039487), *Aldh1* (FBgn0051075), *Jafrac1* (FBgn0040309), *Gclc* (FBgn0040319), *Pgd* (FBgn0004654), *Adh* (FBgn0000055), *HO* (FBgn0037933), *Zw* (FBgn0004057), *Ref(2)P* (FBgn0003231), and *Cyp6g1* (FBgn0025454).

All qRT-PCR experiments were performed in triplicate, and the average of the three replicates in each group was considered. The ∆∆Ct method was used to determine the differences in target gene expression relative to the reference Rp49 gene expression.

### 2.6. Quantitation of GFP- or RFP-Positive Area in CNS

To measure the intensity of GFP fluorescence and the GFP-positive area in the whole larval bodies or the CNSs in the larvae, fluorescence images were acquired using a stereo fluorescence microscope, and the microscopic images were analyzed using Image J software (Ver. 1.54f, National Institutes of Health, Bethesda, MD, USA). The GFP intensity or fluorescence area in the larval CNSs was measured using Fv10i software and quantified using Image J software.

### 2.7. Statistical Analysis

Statistical analyses were performed using GraphPad Prism (Version 9, GraphPad Software, San Diego, CA, USA). The Student’s *t*-test, Welch’s *t*-test, or Pearson’s chi-square test was used to compare the two groups. The F-test, used to determine equal or unequal variance, was initially performed as previously described [45]. *p*-values less than 0.05 were judged to be statistically significant.

## 3. Results

### 3.1. Sesamin Consumption Activated Nrf2/Cnc in Specific Tissues of Drosophila Larvae

**Figure 1:**
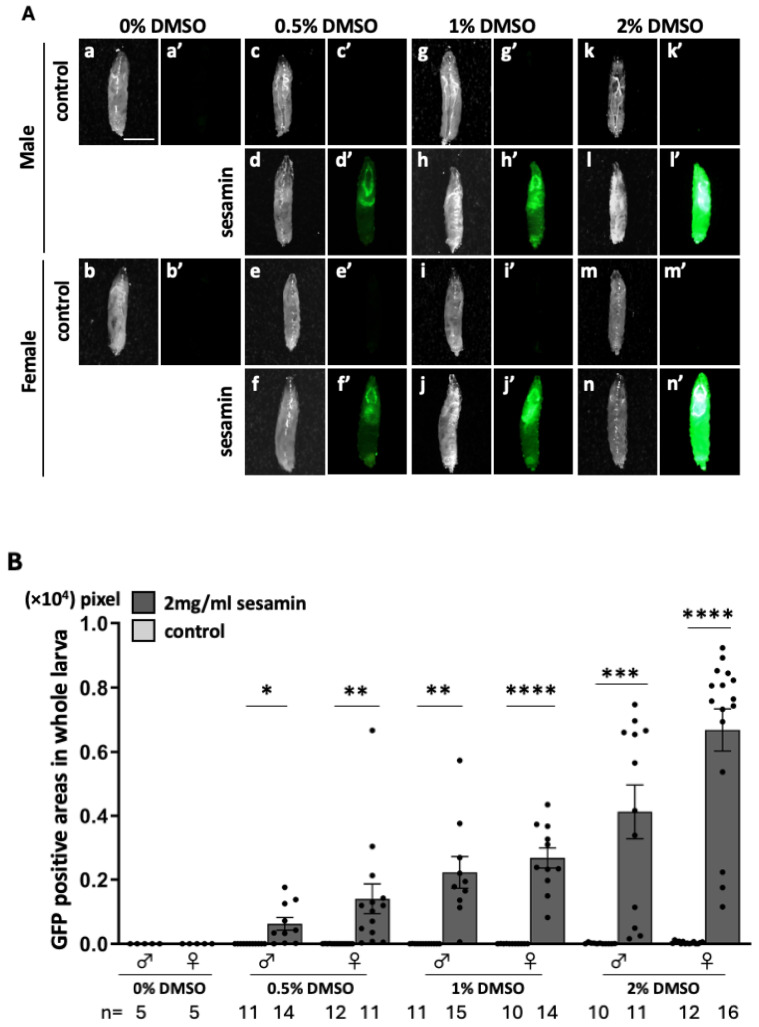
Observation of ARE-GFP fluorescence in the whole bodies of larvae fed sesamin. (**A**) Stereo fluorescence microscopy images of whole-body GFP fluorescence of ARE-GFP larvae. (**a**–**n**) are bright-field stereomicroscopic images of larvae, and (**a’**–**n’**) are fluorescence stereomicroscopic images of the same field of view. Green indicates ARE-GFP fluorescence. The fluorescence is stronger in the larvae fed sesamin (containing 2 mg/mL of sesamin and DMSO) than in the controls (DMSO). Scale bar is 1.0 mm. (**B**) Quantification of GFP-positive regions in whole *ARE-GFP* larvae. The numbers of male and female larvae used in this assay are noted at the bottom. The vertical axis of the graph shows the number of pixels of GFP-positive regions in the whole larval body. * *p* < 0.05, ** *p* < 0.01, *** *p* < 0.001, and **** *p* < 0.0001 with respect to Welch’s *t*-test. Bars indicate SEM.

Based on a previous claim that sesamin-induced Nrf2/Cnc transcription factor activation had an antioxidant effect in *Drosophila* adults [24,25], we investigated whether Nrf2/Cnc was activated in sesamin-fed larvae using an *ARE–GFP* reporter. GFP fluorescence was not observed in the 0.5%, 1%, and 2% DMSO-fed larvae for 24 h (Figure 1A(c’,e’,g’,i’,k’,m’)). The area in which very weak fluorescence could be detected increased as the DMSO concentration increased. The most intense GFP fluorescence was observed in the *ARE–GFP*-expressing larvae that were fed 2 mg/mL of sesamin in 2% DMSO for 24 h (Figure 1A(d’,f’,h’,j’,l’,n’)). GFP fluorescence was observed in the larval head region, indicating that sesamin strongly activated Nrf2/Cnc in the larval tissues. The expression levels of ARE-GFP were quantified. GFP fluorescence was quantified through measuring the GFP-positive regions in whole larval body images (Figure 1B). The positive area increased as the DMSO concentration increased. The largest positive area was observed in the larvae that were fed 2 mg/mL of sesamin in 2% DMSO. The second largest area was observed in the larvae that were fed 1 mg/mL of sesamin in 1% DMSO. In subsequent experiments, we used either feeding condition (2 mg/mL of sesamin in 2% DMSO or 1 mg/mL of sesamin in 1% DMSO) for sesamin administration.

### 3.2. Sesamin Consumption Activated Nrf2/Cnc in the CNS and Digestion-Related Tissues of Drosophila Larvae

**Figure 2:**
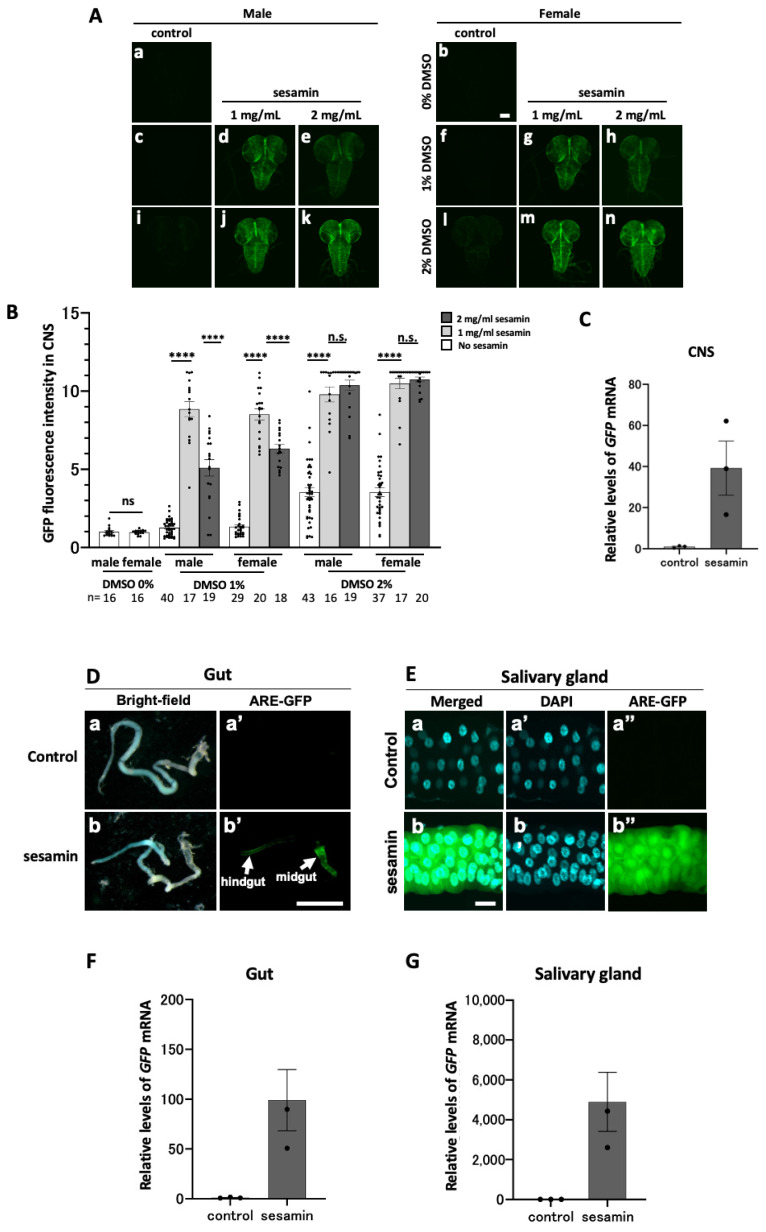
GFP fluorescence of ARE-GFP reporter in the larval CNS and digestion-related tissues and quantitation of *GFP* mRNA in the larvae fed sesamin. (**A**,**D**,**E**) Confocal fluorescence microscopy images of the CNSs (**A**), gut (**D**), and salivary glands (**E**) of ARE-GFP larvae. Note that intense GFP fluorescence was observed in the midgut and hindgut of the sesamin-fed larvae. Green indicates GFP fluorescence. The scale bars indicate 100 µm (**A**,**E**) or 1 mm (**D**). (**B**) Mean (relative) GFP fluorescence intensity in the CNS of *ARE-GFP* larvae. The vertical axis of the graph shows the relative values of the mean GFP fluorescence intensity in the CNSs of larval males reared on the diet without sesamin or DMSO, as a mean value of 1. (**C**,**F**,**G**) Relative expression levels of *GFP* in the CNS (**C**), gut (**F**), or salivary glands (**G**) in female larvae fed the diets supplemented with 1 mg/mL of sesamin (1% DMSO) (control, light-gray bars; sesamin-fed, dark-gray bars) were quantified using qRT-PCR. *GFP* mRNA levels increased from 39- to 4897-fold on average. (**B**) The numbers of male and female larvae used are noted at the bottom. (**C**,**E**,**G**); *n* = 3 (40 larvae/experiment, triplicates). n.s. = not significant and **** indicates *p* < 0.0001 in regard to Student’s *t*-test or Welch’s *t*-test. Bars indicate SEM.

We identified the tissues showing intense GFP fluorescence in the head regions of *ARE-GFP*-expressing larvae after sesamin consumption via examining the presence of sesamin-induced GFP reporter expression in the larval CNS. The late second instar larvae were fed a diet of 1 or 2 mg/mL of sesamin in DMSO and DMSO alone as a control for 24 h. The CNSs of the third instar larvae were observed for GFP fluorescence using confocal microscopy (Figure 2A). GFP fluorescence in the CNS was barely detected in the 1% DMSO-fed larvae (Figure 2A(a–c,f,i,l)), whereas it was relatively higher in the 2% DMSO-fed larvae (Figure 2B). The concentration of DMSO in subsequent experiments was set at 1% because the diet containing 2% DMSO elicited a higher GFP fluorescence intensity in the CNS than that of the 1% DMSO-containing diet. In contrast, the fluorescence intensity in the CNS of the sesamin-fed larvae increased significantly when compared with that of the controls. The highest increase in GFP fluorescence intensity was observed in the larvae fed 2 mg/mL of sesamin (2% DMSO). However, the GFP fluorescence intensity decreased in the CNS of the larvae fed 2 mg/mL of sesamin under 1% DMSO conditions when compared to that for the larvae fed 1 mg/mL of sesamin under the same conditions. Fluorescence intensity fluctuated less in females compared to males. Based on the results of these preliminary experiments, sesamin dissolved in 1% DMSO was mixed with the feed until reaching a concentration of 1 mg/mL and fed to the third instar larvae in the subsequent experiments. Hereafter, this diet is referred to as the sesamin-containing diet. Larvae in the control group were fed a diet containing only 1% DMSO. Hereafter, this diet is referred to as the control diet. Because of the GFP fluorescence difference among the individuals between males and females, female third instar larvae, which showed less of a difference in fluorescence between individuals, were used in subsequent experiments. Next, the expression levels of the *ARE–GFP* reporter in the CNS of the sesamin-containing-diet-fed larvae were quantified using qRT-PCR (Figure 2C). The third instar larval females that consumed a sesamin-containing diet for 24 h showed an average 39.2-fold increase in *GFP* mRNA level when compared with that in the control-diet-fed larvae.

To identify other tissues that show intense GFP reporter expression in the heads of sesamin-fed larvae, we examined whether sesamin induced GFP reporter expression in the salivary glands and larval gut, both of which are located in the larval head, along with the CNS. After the larvae had been consuming the sesamin-containing diet for 24 h, intense GFP fluorescence was observed, particularly in the salivary glands. Stronger GFP fluorescence was observed in the anterior midgut and was less intense in the hindgut. Quantifying the *GFP* mRNA levels using qRT-PCR (with three independent trials for each condition) revealed, on average, 98.9-fold and 4896.6-fold increases in the mRNA levels in the gut and salivary glands, respectively, of the sesamin-fed larvae when compared with those of the control. GFP fluorescence intensity indicates the intensity of Nrf2 activation. Thus, these results indicate that sesamin activates Nrf2 in the larval CNSs, salivary glands, and gut.

### 3.3. Activation of Nrf2/Cnc in Glial Cells of the Larval CNS following Sesamin Consumption

**Figure 3:**
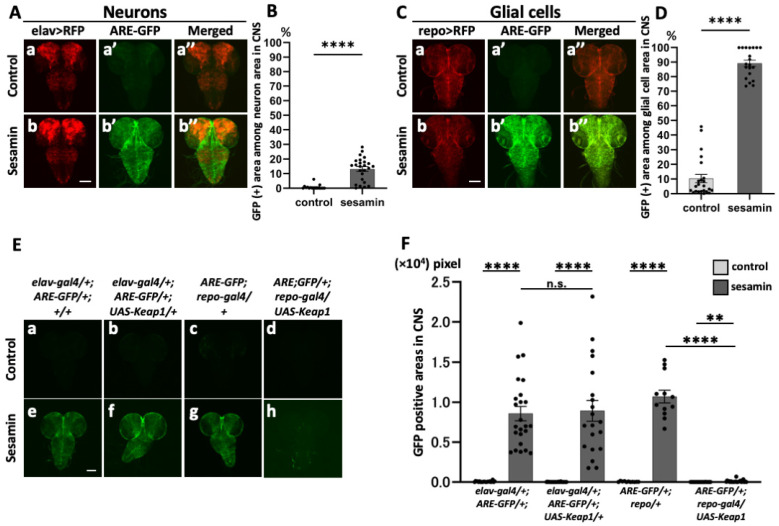
Observation of pan-glial cells within the larval CNS and promotion of its expression by sesamin. (**A**,**C**) Confocal fluorescence microscopy images of GFP fluorescence in all neurons (*elav-Gal4/+*; *ARE-GFP/+*; *UAS-RFP/+* (**A**)) or all glial cells (*ARE-GFP/+*; *repo-Gal4/UAS-RFP* (**C**)) within the CNS of the larvae. GFP fluorescence can be observed in glial cells. Red indicates all neurons or all glial cells, and green indicates ARE-GFP. Scale bar is 100 µm. (**B**,**D**) Percentages of the ARE-GFP region in neurons (**B**) or glial cells (**D**). In both cases, the percentage was significantly higher in the sesamin-fed larvae (*n* = 23 (control), *n* = 20 (sesamin)). (**E**) Confocal fluorescence microscopy images of GFP fluorescence in the CNS in *ARE-GFP* larvae harboring ectopic overexpression of *Keap1* (*elav-Gal4/+*; *ARE-GFP/+*; *UAS-Keap1^EY^/+* (**E**(**b**,**f**)), *ARE-GFP/+*; *repo-Gal4/UAS-Keap1^EY^* (**E**(**d**,**h**))). (**F**) Quantification of GFP-positive regions in the CNS in *ARE-GFP* larvae harboring ectopic overexpression of *Keap1*. GFP regions were fewer in the sesamin-fed larvae overexpressing *Keap1* in all glial cells. ((**F**); *elav-Gal4/+* (control; *n* = 21, sesamin; *n* = 24), *elav-Gal4/+*; *UAS-Keap1^EY^/+* (control; *n* = 26, sesamin; *n* = 20), *repo-Gal4/+* (control; *n* = 11, sesamin; *n* = 20), *repo-Gal4/+*; *UAS-Keap1^EY^/+* (control; *n* = 25, sesamin; *n* = 23); n.s. = not significant, ** *p* < 0.01, and **** *p* < 0.0001 for Student’s *t*-test or Welch’s *t*-test). Bars indicate SEM.

As sesamin induced Nrf2/Cnc activation in the *Drosophila* CNS, we examined whether sesamin activated Nrf2 in the neurons and/or glial cells in the CNS. We labeled all neurons in the CNS based on ectopic RFP expression (*elav-Gal4/+*; *ARE-GFP/+*; *UAS-RFP/+*) and examined *ARE–GFP* reporter expression in the RFP fluorescent areas. Fluorescence was observed throughout the larval brain and ventral ganglia in the CNS of the sesamin-fed larvae, whereas only faint GFP fluorescence was observed in the CNS of the non-sesamin-fed larvae. This GFP-positive region overlapped considerably with the distribution of the neurons. However, intense Nrf2/Cnc activation was also observed in areas with few neurons (Figure 3A,B). In contrast, when all glial cells were labeled with specifically expressing RFP and their distribution in the CNS was observed (*ARE-GFP/+*; *repo-Gal4/UAS-RFP*), the *ARE–GFP* fluorescence was found to approximately coincide with the *repo*-expressing region rather than the *elav*-expressing region after sesamin consumption (Figure 3B,D). Depleting *cnc* causes almost all of the GFP fluorescence to disappear in all cells in *Drosophila* brains with and without sesamin administration [25], although we have not confirmed this in this study. Therefore, the above-mentioned results suggest that strong Nrf2 activation by sesamin occurred more efficiently than in glial cells.

Thus, we confirmed that sesamin activated Nrf2 in glial cells rather than in neurons. We induced the ectopic expression of the Nrf2 inhibitor Keap1 in all neurons and glial cells in the CNS and examined whether *ARE–GFP* fluorescence was lost or reduced in the sesamin-fed larvae. *Keap1* expression was induced in all neurons (*elav-Gal4/+*; *ARE-GFP/+*; *UAS-Keap1^EY^/+*) in the CNS of the larvae that were fed sesamin for 24 h. In the control larvae fed on the diet without sesamin, GFP fluorescence was not observed, confirming the absence of Nrf2 activation (Figure 3E(a–d),F). Although distinctive GFP fluorescence was observed in the CNS of the sesamin-fed larvae (Figure 3E(e),F), the induced expression of *Keap1* in all neurons hardly reduced *ARE–GFP* fluorescence (Figure 3E(f),F). In contrast, intense GFP fluorescence (Figure 3E(g),F) was no longer observed (Figure 3E(h),F) in the sesamin-fed larvae that overexpressed *Keap1* in all glial cells of the CNS (*ARE-GFP/+*; *repo-Gal4/UAS-Keap1^EY^*). These results suggest that Keap1 can repress the activation of Nrf2 in glial cells, while this is not the case in neurons.

### 3.4. Sesamin Activated Nrf2 in the Astrocytes, Cortex, and Surface Glia

**Figure 4:**
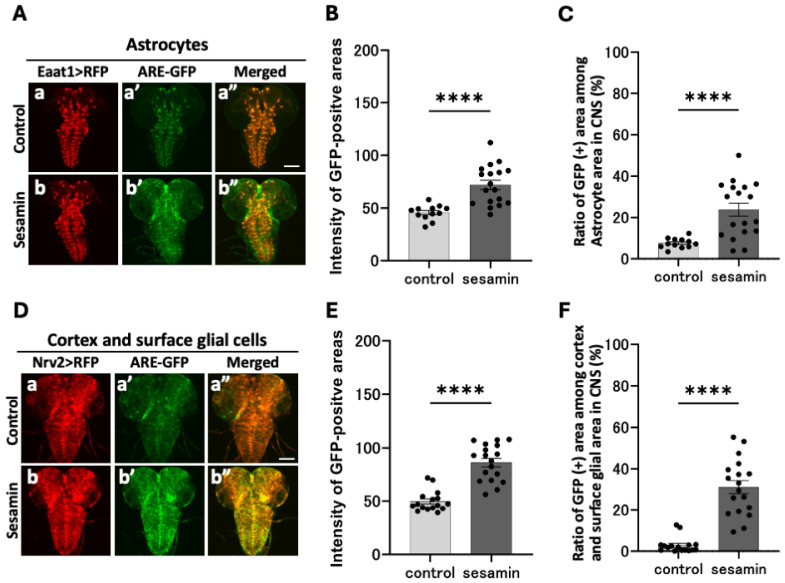
Observation of ARE-GFP in specific glial cells within the CNS of larvae and its expression enhanced by sesamin. (**A**,**D**) Confocal fluorescence microscopy images of GFP fluorescence in the glial cells in the CNS of *Eaat1-Gal4/ARE-GFP*; *UAS-RFP/+* larvae (**A**) or *Nrv2-Gal4/ARE-GFP*; *UAS-RFP/+* larvae (**D**). The images are dorsal (**A**) and ventral views of the CNS, respectively. Red indicates astrocytes (**A**) or cortex and surface glial cells, and green indicates ARE-GFP. The scale bar is 100 µm. (**B**,**E**) The intensity of the GFP fluorescence in and around astrocytes (**B**) or cortex and surface glial cells (**E**) (quantified from 0 to 255). The intensity in each glial cell was significantly higher in the larvae fed sesamin compared to that of the controls. (**C**,**F**) Percentages of ARE-GFP regions in astrocytes or cortex and surface glial cells. In each glial cell, the percentage was significantly increased when compared to that of the controls (*n* = 12 (control), *n* = 18 (sesamin) (**B**,**C**), *n* = 16 (control), and *n* = 18 (sesamin) (**E**,**F**), with **** indicating *p* < 0.0001, for Welch’s *t*-test). Bars indicate SEM.

As previously mentioned, sesamin activates the Nrf2 transcription factor in glial cells within the larval CNS. Next, we investigated the types of glial cells in which Nrf2 is activated. Using the Gal4/UAS system, which allows ectopic gene expression, we performed the astrocyte (Figure 4A(a,b)), *Eaat1-Gal4/ARE-GFP*; *UAS-RFP/+*)- or cortex- and surface glia (Figure 4D(a,b), *Nrv2-Gal4/ARE-GFP*; *UAS-RFP/+*)-specific RFP labeling of the glial cells within the CNS. After feeding the larvae sesamin for 24 h, we examined whether the RFP fluorescence areas indicating each type of glial cell overlapped with those of the Nrf2-activated cells in the CNS. Greater GFP fluorescence was observed in the CNS of the sesamin-fed larvae (Figure 4A(b’)) than in the controls (Figure 4A(a’)). GFP fluorescence intensity in each glial cell region was quantified on a 0–255 scale. The average fluorescence intensity in the sesamin-fed larvae was 72.0 (57.4% increase); for comparison, it was 45.7 in the controls (Figure 4B). Similarly, in the cortex and surface glial regions, the average fluorescence intensity in the sesamin-fed group was 86.2 (73.2% increase, Figure 4D(b’),E), while it was 49.8 in the controls (Figure 4D(a’),E). As the fluorescence detected in the CNS of the controls was weak, we measured the sizes of the positive areas that expressed each glia marker with greater GFP fluorescence intensity than the average fluorescence intensity of the controls (which were not fed sesamin). The lower limit of the fluorescence threshold was set as the average fluorescence intensity of the controls (this threshold was 46–255 for astrocytes and 50–255 for cortex and surface glia). We observed greater GFP fluorescence in the sesamin-fed larvae than in the controls (Figure 4B). Moreover, we found that 23.8% of astrocytes in the sesamin-fed larvae emitted fluorescence, which is a 16.2% increase in the fluorescence-expressing area compared with that of the controls, for which the figure was 7.6% (Figure 4C). Among the cortex and surface glial cells of the sesamin-fed larvae, 31.1% of fluorescence-emitting regions were detected, which was a 28.2% increase when compared with the 2.9% detected in the controls (Figure 4F). These results indicate that sesamin consumption activates Nrf2 in astrocytes as well as in cortex and surface glial cells.

### 3.5. Sesamin Consumption in Larvae Elevated the mRNA Levels of Several Genes Encoding Enzymes in Cytochrome P450

Using the *ARE–GFP* reporter, we showed that sesamin consumption induced a pronounced upregulation of Nrf2-dependent transcription in the CNS, salivary glands, and gut. This GFP reporter has an artificial regulatory region harboring up to four consecutive ARE sequences aligned in tandem before the *GFP* cDNA, causing GFP to be excessively expressed in response to Nrf2 activation. To quantify the upregulation of the Nrf2-targeted endogenous genes, we performed quantitative PCR using RNA prepared from the CNSs of sesamin-fed larvae. The mRNA levels of the *GFP* gene transcribed from *ARE–GFP* increased 40-fold on average when compared to that in the control larvae reared on a diet without sesamin (Figure 2C).

**Figure 5:**
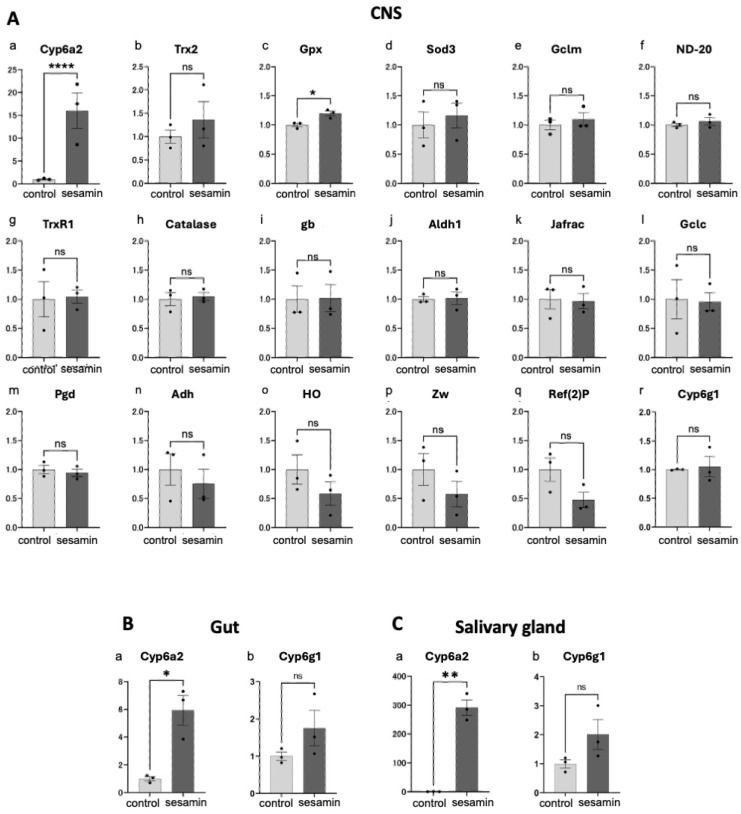
Quantification of mRNA for Nrf2-regulated genes in the CNSs and digestive-related tissues in larvae reared on sesamin. (**A**) Relative expression levels of each gene in larvae fed sesamin (control, light-gray bars, sesamin, dark-gray bars) were quantified using qRT-PCR. Total RNA was isolated from the CNSs (**A**), gut (**B**), or salivary glands (**C**) of larvae reared on diets containing 1 mg/mL sesamin and 1% DMSO, and those from larvae reared on diets containing 1% DMSO only as controls for 24 h. *Cyp6a2* mRNA levels were significantly increased in the CNS, gut, and salivary glands in the larvae fed sesamin (*n* = 3). (ns, not significant; * *p* < 0.05; ** *p* < 0.01; and **** *p* < 0.0001 for Welch’s *t*-test). Bars indicate SEM.

Next, we attempted to identify the endogenous genes whose transcription was induced by sesamin. Only a few Nrf2/Cnc target genes have been identified in *Drosophila*. Among the known Nrf2 target genes in mammals, we selected 17 genes whose orthologues were conserved in *Drosophila* as candidates for Nrf2 targets [46]. qRT-PCR was performed to quantify the target mRNA levels in the CNS of the third instar larvae (40 larvae/experiment) that were fed diets supplemented with sesamin or only 1% DMSO (control) for 24 h using synthesized cDNAs as templates (Figure 5A). Among these 17 genes, the mRNA level of the gene encoding Cyp6a2 (Figure 5A(a)), which is a member of the cytochrome p450 family, increased 16.0-fold in the CNS of the sesamin-fed larvae when compared with that of the controls. Among the remaining 16 candidate Nrf2 target genes, the average mRNA levels of *Trx2* (Figure 5A(b)), *Gpx* (Figure 5A(c)), and *Sod3* (Figure 5A(d)) were slightly higher than those of the controls (constituting 36.0%, 19.6%, and 16.0% increases, respectively), although the differences for *Trx2* and *Sod3* from the controls were not statistically significant. The mRNA levels of nine other genes, namely, *Gclm* (Figure 5A(e)), *ND-20/Nqo1* (Figure 5A(f)), *TrxR1* (Figure 5A(g)), *Catalase* (Figure 5A(h)), *gb* (Figure 5A(i)), *Aldh1* (Figure 5A(j)), *Jafrac1* (Figure 5A(k)), *Gclc* (Figure 5A(l)), *Pgd* (Figure 5A(m)), and *Cy6g1* (Figure 5A(r)), remained essentially unchanged after the larvae were fed (<10% change in each case). In contrast, the mRNA levels of *Adh* (Figure 5A(n)), *HO* (Figure 5A(o)), *Zw* (Figure 5A(p)), and *Ref2(2)p*/*p62* (Figure 5A(q)) were reduced (24.7%, 41.6%, 42.4%, and 52.5% reductions, respectively), although the differences for these genes from controls were not statistically significant.

As sesamin significantly increased the *Cyp6a2* mRNA levels in the larval CNS, we investigated whether its mRNA levels were altered in other larval tissues involved in absorption and metabolism, such as the gut and salivary gland. The *Cyp6a2* mRNA levels in the gut and salivary glands of the sesamin-fed larvae showed significant 5.9-fold (Figure 5B(a)) and 291.7-fold (Figure 5C(a)) increases when compared with those of the controls. *Drosophila Cyp6a2* and *Cyp6g1* encode enzymes involved in insecticide metabolism. Both genes are orthologs of human cytochrome P450 family 3 subfamily A member 43 (Cyp3a4). Whether these genes are Nrf2 targets is unknown; nevertheless, we investigated whether sesamin consumption increased its expression. Consequently, the mRNA levels of *Cyp6g1* in the gut and salivary glands of sesamin-fed larvae were also 1.8-fold (Figure 5B(b)) and 2.0-fold (Figure 5C(b)) higher than those in the controls. In summary, the average mRNA levels of two genes, *Cyp6a2* and *Cyp6g1*, which encode cytochrome P450 enzymes, were higher in tissues related to the *Drosophila* larval digestive system after sesamin consumption than those in controls, although the difference in *Cyp6g1* from the control was not statistically significant.

### 3.6. Resistance to the Neonicotinoid Insecticide Imidacloprid Was Not Observed in Sesamin-Fed Larvae

The mRNA levels of the cytochrome P450 drug-metabolizing enzyme Cyp6a2 increased notably in the CNS, gut, and salivary glands of the sesamin-fed larvae. A similar drug-metabolizing enzyme, Cyp6g1, was also induced in the gut and salivary glands of the larvae, although the difference from the control was not statistically significant. Cyp6g1 is involved in the metabolism of neonicotinoids that have toxic effects on neuronal connections in *Drosophila* [47], and we predicted that the accelerated metabolism of this chemical would eventually lead to its rapid degradation in organisms, potentially inducing drug resistance. Therefore, we investigated whether larvae reared on sesamin exhibit resistance to the neonicotinoid insecticide imidacloprid. First, we determined the suitable concentration of imidacloprid for the study by examining the pupation rates. *Drosophila* larvae failed to pupate or were killed at the larval stage (survival rate 30%) at insecticide concentrations in the range of 3–100 µM. The pupation rate was 100% (16 out of 16 larvae) for larvae fed the standard diet without the drug. In contrast, the pupation rates were 31.1% (14 of 45 larvae) for 3 µM, 31.0% (13 of 42 larvae) for 10 µM, 28.6% (10 of 35 larvae) for 30 µM, and 0% (0 out of 19 larvae) for 100 µM of the drug. An imidacloprid concentration of 3 µM was selected for the subsequent experiments as the corresponding survival rate was approximately 30%. To induce *Cyp6a2* and *Cyp6g1* expression in advance, the standard cornmeal food supplemented with 1 mg/mL of sesamin and 1% DMSO was fed to the first instar larvae before drug administration. Parent flies were placed in vials while being fed the sesamin-containing or control diets for 8 h and allowed to lay eggs. After removing the parent flies, the hatched larvae were reared on the same diet for 72 h. The second instar larvae (50 larvae) were transferred to a diet consisting of 3 µM of imidacloprid and 1 mg/mL of sesamin with 1% DMSO (the control diet contained 3 µM of imidacloprid and 1% DMSO) from the second instar stage until they pupated. We found that 26.8 ± 2.7% of the larvae (67 of 250 larvae) that were fed diets containing sesamin, DMSO, and 3 µM of imidacloprid pupated. In contrast, the pupation rate was 27.6 ± 4.9% (69 of 250) among the controls. However, no significant differences were observed between the two groups (*p* > 0.05). These results indicate that the induction of *Cyp6a2* and *Cyp6g1* expression by sesamin in the digestion-related tissues did not confer resistance to 3 µM imidacloprid.

## 4. Discussion

### 4.1. Activation of Nrf2/Cnc in Specific Larval Tissues by Sesamin

Sesamin is an abundantly occurring compound in sesame seeds. Its health benefits, such as its antioxidant [27], antihypertensive [48], and alcohol-metabolizing [49] properties, have been previously reported. Previous studies have revealed that sesamin exerts its antioxidant effects on *Drosophila* adults by activating the Nrf2/Cnc transcription factor, which is required for oxidative stress response [24]. Moreover, Nrf2 activation may be caused by the inhibition of Nrf2–Keap1 binding by sesamin [25]. Additionally, sesamin activates Nrf2/Cnc in larval tissues. Strong Nrf2 activation was observed in the CNS, salivary glands, and gut, including the upper midgut and hindgut. These results are consistent with those of previous studies which showed that sesamin activates Nrf2 in the adult brain and gut [24]. The activation of Nrf2 in intestinal epithelial cells by sesamin may be transmitted to the CNS via neural networks. *Drosophila* enteric nerves connect to the following two regions: the region consisting of the foregut, proventriculus, and upper midgut, and the hindgut (the majority of which has no nerve extensions or connections), which is the largest region in the intestinal tract [50,51]. Therefore, *Drosophila* intestinal epithelial cells, in which Nrf2 is activated during the absorption of sesamin, may transmit signals to the CNS via neural networks. Alternatively, sesamin may act directly on the CNS via the hemolymph after absorption by intestinal epithelial cells. In the mammalian liver, sesamin is metabolized into polyphenol-like metabolite SC1, which is present as an intermediate product [52]. Sesamin is also metabolized into SC1 in the gut epithelial cells of *Drosophila* adults [24]. It is metabolized into SC1 in intestinal epithelial cells, absorbed, released into the larval hemolymph, and transported to the CNS through the larval hemolymph. In mammals, a rigid barrier known as the blood–brain barrier prevents most drugs and other substances in the blood from entering the brain [53]. In contrast, the blood–brain barrier in *Drosophila* is relatively more permeable than that in mammals [54]. Therefore, SCI, which is a water-soluble metabolite of sesamin, may enter the CNS directly via the hemolymph without needing to pass through a neural network connected to the gut cells. We showed that sesamin consumption induced higher Nrf2 activation in the CNS, gut, and salivary gland, but not in other larval tissues. This variation in the degree of Nrf2 activation by sesamin among larval tissues could be attributed to variations in the sensitivity to sesamin or the efficiency of its uptake in different tissues. Hence, the mechanisms underlying the cell-specific effects of sesamin need to be clarified in future studies.

### 4.2. Activation of Nrf2/Cnc in Larval Glial Cells in CNS by Sesamin

This study demonstrated intense Nrf2 activation in glial cells within the CNS of sesamin-fed larvae. The ectopic expression of Keap1, which is an Nrf2 inhibitor, in glial cells repressed most sesamin-induced Nrf2 activation in the CNS. However, Nrf2 induction in the neurons did not change to a significant extent. This suggests that Nrf2 activation in the CNS occurs more efficiently than in glial cells. Glial cells are intimately involved in the physiological actions of the entire CNS, including developmental and nutritional processes, the functioning of the immune system, and the electrical insulation of neurons [55,56,57]. Glial cells are as crucial as neurons in maintaining brain integrity and function [58]. Glial cell dysfunction is closely associated with various neurological disorders such as autism, schizophrenia, and neurodegeneration. Nrf2 activation in glial cells suppresses the Parkinson’s phenotype in a *Drosophila* disease model [59]. Nrf2 activation in the glial cells rather than in the neurons had a greater protective effect against oxidative stress in the CNS. In this study, sesamin strongly activated Nrf2 in the glial cells of the larval CNS. Sesamin consumption activates Nrf2 in specific neurons, such as glutamatergic, cholinergic, and dopaminergic neurons, in the adult brain. Furthermore, it suppresses neuronal loss associated with oxidative stress accumulation [24,25]. Although Nrf2 activation in the glial cells of the adult brain was not examined in this study, the neuroprotective effect of sesamin may be reasonably attributed to the activation of Nrf2 in glial cells associated with neurons in the adult *Drosophila* brain. The Nrf2-dependent expression of antioxidative genes in glial cells may improve the oxidative condition of neurons, which could maintain neuronal homeostasis in the CNS.

The activation of NF-κB-transcription factor-mediated signaling in innate immune pathways was accelerated with oxidative stress and vascular inflammation occurring during aging in the rats [60]. During the neuroinflammation observed in neurodegenerative disease models, the microglia are activated and secrete excessive quantities of cytokines that stimulate astrocytes to secrete extracellular factors that then induce neuronal cell death [61]. Recently, the production of neuronal death-inducing factors in astrocytes was reported to be suppressed by inhibiting the NF-κB pathway via Nrf2 activation in astrocytes [62]. We found that sesamin activates Nrf2 in astrocytes within the larval CNS. Therefore, sesamin-induced Nrf2 activation in astrocytes may suppress neurodegeneration by suppressing excess cytokine production in the astrocytes. We plan to study this further in future studies. Thus, sesamin is a promising health-promoting agent that suppresses the progression of human neurodegenerative diseases and alleviates their symptoms.

### 4.3. Induction of Cytochrome P450 Drug-Metabolizing Gene Expression in the Larval CNS and Digestion-Related Tissues by Sesamin and Its Effects on Organisms

This study demonstrates that sesamin consumption significantly increases the mRNA level of *Cyp6a2* in the larval CNS and salivary glands and that there is a tendency for it to increase the level of *Cyp6g1* in the salivary glands and gut. The *Cyp6a2* gene is an ortholog of the human *CYP3A4* gene, which encodes an enzyme that is involved in the metabolism of hypnotic sedatives, antidepressants, and antiepileptic drugs. Cyp6g1 can also metabolize dichlorodiphenyltrichloroethane (DDT) and imidacloprid, which are used as pesticides [63]. Furthermore, *Drosophila* wild-type stocks that show resistance to DDT also exhibit the elevated expression of *Cyp6g1* and *Cyp6a2* genes [42,43]. Thus, the sesamin-induced elevated expression of these genes could imply accelerated drug metabolism; that is, the drug would be rapidly degraded in the body. The sesamin-administered larvae were fed imidacloprid to examine whether resistance was conferred against the pesticides. Contrary to expectations, no significant changes were observed in drug resistance when compared with that of the controls. As amino acid substitution in Cyp6a2 was observed when reporting altered activity in resistant stocks [64], increased Cyp6a2 expression, such as that observed in this study, may not be sufficient to impart resistance; alternatively, the elevated expression levels of the two cytochrome P450 genes could have been insufficient. Additionally, we cannot neglect the fact that the imidacloprid concentration used in this study was considerably high; hence, the sesamin-induced increase in *Cyp6a2* and *Cyp6g1* expression may be insufficient to eliminate a substantial concentration of the toxin. Neonicotinoids, including imidacloprid, disrupt insect neurotransmission by binding to nicotinic acetylcholine receptors (nAchR). Although the effects of these toxins on mammals have not been fully elucidated, they are known to adversely affect the human brain—particularly with respect to neural development [65]. The present study found that sesamin activates Nrf2 in the developing larval CNS. Therefore, if the detoxifying effects of sesamin on neonicotinoids can be verified, similar adverse effects on human neural development may be possibly prevented. DDT is absorbed through the insect epidermis and acts on Na^+^ channels to reach the nerve terminals. DDT is metabolized in the body into highly bioaccumulating metabolites. Although these pesticides are no longer used, they remain bioaccumulated in the environment. They are associated with the pathogenesis of certain types of cancers and Alzheimer’s disease [66,67]. DDT is difficult to metabolize and excrete, but the Cyp6g1 and Cyp6a2 produced in DDT-resistant mutants may metabolize and detoxify this chemical. Sesamin would provide a new utility if it could promote DDT detoxification by upregulating drug-metabolizing enzymes. Therefore, the effect of sesamin on DDT detoxification should be investigated in future studies.

## Data Availability

The study did not report any data.

## References

[1] Kawanishi S., Hiraku Y., Oikawa S. (2001). Mechanism of guanine-specific DNA damage by oxidative stress and its role in carcinogenesis and aging. Mutat. Res..

[2] Glei M., Schaeferhenrich A., Claussen U., Kuechler A., Liehr T., Weise A., Marian B., Sendt W., Pool-Zobel B.L. (2007). Comet fluorescence in situ hybridization analysis for oxidative stress–induced DNA damage in colon cancer relevant genes. Toxicol. Sci..

[3] Chandrasekaran A., del Pilar Sosa Idelchik M., Melendez J.A. (2016). Redox control of senescence and age-related disease. Redox Biol..

[4] Hedge M., Lortz S., Drinkgern J., Lenzen S. (1997). Relation between antioxidant enzyme gene expression and antioxidative defense status of insulin-producing cells. Skelet. Muscle.

[5] Kirkman H.N., Rolfo M., Ferraris A.M., Gaetani G.F. (1999). Mechanisms of protection of catalase by NADPH: Kinetics and stoichiometry. J. Biol. Chem..

[6] Hirotsu Y., Katsuoka F., Funayama R., Nagashima T., Nishida Y., Nakayama K., Engel J.D., Yamamoto M. (2012). Nrf2-MafG heterodimers contribute globally to antioxidant and metabolic networks. Nucleic Acids Res..

[7] Moi P., Chan K., Asunis I., Cao A., Kan Y.W. (1994). Isolation of NF-E2-related factor 2 (Nrf2), a NF-E2-like basic leucine zipper transcriptional activator that binds to the tandem NF-E2/AP1 repeat of the beta-globin locus control region. Proc. Natl. Acad. Sci. USA.

[8] Venugopal R., Jaiswal A.K. (1996). Nrf1 and Nrf2 positively and c-Fos and Fra1 negatively regulate the human antioxidant response element-mediated expression of NAD(P)H:quinone oxidoreductase1 gene. Proc. Natl. Acad. Sci. USA.

[9] Itoh K., Chiba T., Takahashi S., Ishii T., Igarashi K., Katoh Y., Oyake T., Hayashi N., Satoh K., Hatayama I. (1997). An Nrf2/Small Maf heterodimer mediates the induction of phase II detoxifying enzyme genes through antioxidant response elements. Biochem. Biophys. Res. Commun..

[10] McMahon M., Swift S.R., Hayes J.D. (2018). Zinc-binding triggers a conformational-switch in the cullin-3 substrate adaptor protein KEAP1 that controls transcription factor NRF2. Toxicol. Appl. Pharmacol..

[11] Kobayashi A., Kang M.-I., Watai Y., Tong K.I., Shibata T., Uchida K., Yamamoto M. (2006). Oxidative and electrophilic stresses activate Nrf2 through inhibition of Ubiquitination activity of Keap1. Mol. Cell. Biol..

[12] Chatterjee N., Bohmann D. (2012). A versatile ΦC31 based reporter system for measuring AP-1 and Nrf2 signaling in Drosophila and in tissue culture. PLoS ONE.

[13] Dinkova-Kostova A.T., Holtzclaw W.D., Cole R.N., Itoh K., Wakabayashi N., Katoh Y., Yamamoto M., Talalay P. (2002). Direct evidence that sulfhydryl groups of Keap1 are the sensors regulating induction of phase 2 enzymes that protect against carcinogens and oxidants. Proc. Natl. Acad. Sci. USA.

[14] Furukawa M., Xiong Y. (2005). BTB protein Keap1 targets antioxidant transcription factor Nrf2 for ubiquitination by the Cullin 3-Roc1 ligase. Mol. Cell Biol..

[15] Hartenstein V., Tepass U., Gruszynski-Defeo E. (1994). Embryonic development of the stomatogastric nervous system in Drosophila. J. Comp. Neurol..

[16] Sakurai T., Kanayama M., Shibata T., Itoh K., Kobayashi A., Yamamoto M., Uchida K. (2006). Ebselen, a seleno-organic antioxidant, as an electrophile. Chem. Res. Toxicol..

[17] Rachakonda G., Xiong Y., Sekhar K.R., Stamer S.L., Liebler D.C., Freeman M.L. (2008). Covalent modification at Cys151 dissociates the electrophile sensor Keap1 from the ubiquitin ligase CUL3. Chem. Res. Toxicol..

[18] Shih P.-H., Yen G.-C. (2007). Differential expressions of antioxidant status in aging rats: The role of transcriptional factor Nrf2 and MAPK signaling pathway. Biogerontology.

[19] Schmidlin C.J., Dodson M.B., Madhavan L., Zhang D.D. (2019). Redox regulation by NRF2 in aging and disease. Free Radic. Biol. Med..

[20] Andargie M., Vinas M., Rathgeb A., Möller E., Karlovsky P. (2021). Lignans of sesame (*Sesamum indicum* L.): A Comprehensive Review. Molecules.

[21] Akimoto K., Kitagawa Y., Akamatsu T., Hirose N., Sugano M., Shimizu S., Yamada H. (1993). Protective effects of sesamin against liver damage caused by alcohol or carbon tetrachloride in rodents. Ann. Nutr. Metab..

[22] Ashakumary L., Rouyer I., Takahashi Y., Ide T., Fukuda N., Aoyama T., Hashimoto T., Mizugaki M., Sugano M. (1999). Sesamin, a sesame lignan, is a potent inducer of hepatic fatty acid oxidation in the rat. Metab. Clin. Exp..

[23] Li X., Gao Y., Li S., Yang J. (2015). Effect of sesamin on pulmonary vascular remodeling in rats with monocrotaline-induced pulmonary hypertension. China J. Chin. Mater. Medica.

[24] Le T.D., Nakahara Y., Ueda M., Okumura K., Hirai J., Sato Y., Takemoto D., Tomimori N., Ono Y., Nakai M. (2019). Sesamin suppresses aging phenotypes in adult muscular and nervous systems and intestines in a Drosophila senescence-accelerated model. Eur. Rev. Med. Pharmacol. Sci..

[25] Le T.D., Inoue Y.H. (2021). Sesamin Activates Nrf2/Cnc-dependent transcription in the absence of oxidative stress in Drosophila adult brains. Antioxidants.

[26] Ikeda T., Nishijima Y., Shibata H., Kiso Y., Ohnuki K., Fushiki T., Moritani T. (2003). Protective effect of sesamin administration on exercise-induced lipid peroxidation. Int. J. Sports Med..

[27] Nakai M., Harada M., Nakahara K., Akimoto K., Shibata H., Miki W., Kiso Y. (2003). Novel antioxidative metabolites in rat liver with ingested sesamin. J. Agric. Food Chem..

[28] Kiso Y. (2004). Antioxidative roles of sesamin, a functional lignan in sesame seed, and it’s effect on lipid- and alcohol-metabolism in the liver: A DNA microarray study. BioFactors.

[29] Lee W.J., Ou H.C., Wu C.M., Lee I.T., Lin S.Y., Lin L.Y., Tsai K.L., Lee S.D., Sheu W.H.H. (2009). Sesamin mitigates inflammation and oxidative stress in endothelial cells exposed to oxidized low-density lipoprotein. J. Agric. Food Chem..

[30] Takemoto D., Yasutake Y., Tomimori N., Ono Y., Shibata H., Hayashi J. (2015). Sesame lignans and vitamin E supplementation improve subjective statuses and anti-oxidative capacity in healthy humans with feelings of daily fatigue. Glob. J. Health Sci..

[31] Hagen T.M. (2003). Oxidative Stress, Redox Imbalance, and the Aging Process. Antioxid. Redox Sign..

[32] Stevenson R., Samokhina E., Rossetti I., Morley J.W., Buskila Y. (2020). Neuromodulation of glial function during neurodegeneration. Front. Cell Neurosci..

[33] Martin C.A., Krantz D.E. (2014). *Drosophila melanogaster* as a genetic model system to study neurotransmitter transporters. Neurochem. Int..

[34] Lessing D., Bonini N.M. (2009). Maintaining the brain: Insight into human neurodegeneration from Drosophila melanogaster mutants. Nature Rev. Genet..

[35] Nichols C.D. (2006). *Drosophila melanogaster* neurobiology, neuropharmacology, and how the fly can inform CNS drug discovery. Pharmacol. Therapeut..

[36] Rera M., Clark R.I., Walker D.W. (2012). Intestinal barrier dysfunction links metabolic and inflammatory markers of aging to death in Drosophila. Proc. Natl. Acad. Sci. USA.

[37] Miguel-Aliaga I., Jasper H., Lemaitre B. (2018). Anatomy and physiology of the digestive tract of *Drosophila melanogaster*. Genetics.

[38] Janssen A.W.F., Duivenvoorde L.P.M., Rijkers D., Nijssen R., Peijnenburg A.A.C.M., van der Zande M., Louisse J. (2021). Cytochrome P450 expression, induction and activity in human induced pluripotent stem cell-derived intestinal organoids and comparison with primary human intestinal epithelial cells and Caco-2 cells. Arch. Toxicol..

[39] Ashino T., Ohkubo-Morita H., Yamamoto M., Yoshida T., Numazawa S. (2014). Possible involvement of nuclear factor erythroid 2-related factor 2 in the gene expression of Cyp2b10 and Cyp2a5. Redox Biol..

[40] Lämsä V., Levonen A.L., Leinonen H., Ylä-Herttuala S., Yamamoto M., Hakkola J. (2010). Cytochrome P450 2A5 constitutive expression and induction by heavy metals is dependent on redox-sensitive transcription factor Nrf2 in liver. Chem. Res. Toxicol..

[41] Ashino T., Yamamoto M., Numazawa S. (2020). Nrf2 antioxidative system is involved in cytochrome P450 gene expression and activity: A delay in pentobarbital metabolism in Nrf2-deficient mice. Drug Metab. Dispos..

[42] Daborn P., Boundy S., Yen J., Pittendrigh B., Ffrench-Constant R. (2001). DDT resistance in Drosophila correlates with Cyp6g1 over-expression and confers cross-resistance to the neonicotinoid imidacloprid. Mol. Genet. Genom..

[43] Pedra J.H., McIntyre L.M., Scharf M.E., Pittendrigh B.R. (2004). Genome-wide transcription profile of field- and laboratory-selected dichlorodiphenyltrichloroethane (DDT)-resistant Drosophila. Proc. Natl. Acad. Sci. USA.

[44] Oka S., Hirai J., Yasukawa T., Nakahara Y., Inoue Y.H. (2015). A correlation of reactive oxygen species accumulation by depletion of superoxide dismutases with age-dependent impairment in the nervous system and muscles of Drosophila adults. Biogerontology.

[45] Kinoshita S., Takarada K., Kinoshita Y., Inoue Y.H. (2022). Drosophila hemocytes recognize lymph gland tumors of mxc mutants and activate the innate immune pathway in a reactive oxygen species-dependent manner. Biol. Open.

[46] Ma Q. (2013). Role of Nrf2 in oxidative stress and toxicity. Ann. Rev. Pharmacol..

[47] Fusetto R., Denecke S., Perry T., O’Hair R.A.J., Batterham P. (2017). Partitioning the roles of CYP6G1 and gut microbes in the metabolism of the insecticide imidacloprid in *Drosophila melanogaster*. Sci. Rep..

[48] Nakano D., Kwak C.J., Fujii K., Ikemura K., Satake A., Ohkita M., Takaoka M., Ono Y., Nakai M., Tomimori N. (2006). Sesamin metabolites induce an endothelial nitric oxide-dependent vasorelaxation through their antioxidative property-independent mechanisms: Possible involvement of the metabolites in the antihypertensive effect of sesamin. J. Pharmacol. Exp. Ther..

[49] Tsuruoka N., Kidokoro A., Matsumoto I., Abe K., Kiso Y. (2005). Modulating effect of sesamin, a functional lignan in sesame seeds, on the transcription levels of lipid- and alcohol-metabolizing enzymes in rat liver: A DNA microarray study. Biosci. Biotechnol. Biochem..

[50] Spiess R., Schoofs A., Heinzel H.-G. (2008). Anatomy of the stomatogastric nervous system associated with the foregut in *Drosophila melanogaster* and *Calliphora vicina* third instar larvae. J. Morphol..

[51] Cognigni P., Bailey A.P., Miguel-Aliaga I. (2011). Enteric neurons and systemic signals couple nutritional and reproductive status with intestinal homeostasis. Cell Metab..

[52] Yasuda K., Ikushiro S., Kamakura M., Ohta M., Sakaki T. (2010). Metabolism of sesamin by cytochrome P450 in human liver microsomes. Drug Metab. Dispos..

[53] Pardridge W.M. (2003). Blood-brain barrier drug targeting: The future of brain drug development. Mol. Interv..

[54] Hindle S.J., Bainton R.J. (2014). Barrier mechanisms in the Drosophila blood-brain barrier. Front. Cell Neurosci..

[55] Ullian E.M., Sapperstein S.K., Christopherson K.S., Barres B.A. (2001). Control of synapse number by glia. Science.

[56] Constantinescu C.S., Tani M., Ransohoff R.M., Wysocka M., Hilliard B., Fujioka T., Murphy S., Tighe P.J., Das Sarma J., Trinchieri G. (2005). Astrocytes as antigen-presenting cells: Expression of IL-12/IL-23. J. Neurochem..

[57] Edgar J.M., Garbern J. (2004). The myelinated axon is dependent on the myelinating cell for support and maintenance: Molecules involved. J. Neurosci. Res..

[58] Edenfeld G., Stork T., Klämbt C. (2005). Neuron-glia interaction in the insect nervous system. Curr. Opin. Neurobiol..

[59] Guo Q., Wang B., Wang X., Smith W.W., Zhu Y., Liu Z. (2021). Activation of Nrf2 in astrocytes suppressed PD-Like phenotypes via antioxidant and autophagy pathways in rat and *Drosophila* models. Cells.

[60] Ungvari Z., Orosz Z., Labinskyy N., Rivera A., Xiangmin Z., Smith K., Csiszar A. (2007). Increased mitochondrial H_2_O_2_ production promotes endothelial NF-κB activation in aged rat arteries. Am. J. Physiol. Heart Circ. Physiol..

[61] Liddelow S.A., Guttenplan K.A., Clarke L.E., Bennett F.C., Bohlen C.J., Schirmer L., Bennett M.L., Münch A.E., Chung W.-S., Peterson T.C. (2017). Neurotoxic reactive astrocytes are induced by activated microglia. Nature.

[62] Nakano-Kobayashi A., Canela A., Yoshihara T., Hagiwara M. (2023). Astrocyte-targeting therapy rescues cognitive impairment caused by neuroinflammation via the Nrf2 pathway. Proc. Natl. Acad. Sci. USA.

[63] Joußen N., Heckel D.G., Haas M., Schuphan I., Schmidt B. (2008). Metabolism of imidacloprid and DDT by P450 CYP6G1 expressed in cell cultures of Nicotiana tabacum suggests detoxification of these insecticides in Cyp6g1-overexpressing strains of *Drosophila melanogaster*, leading to resistance. Pest Manag. Sci..

[64] Amichot M., Tarès S., Brun-Barale A., Arthaud L., Bride J.M., Bergé J.B. (2004). Point mutations associated with insecticide resistance in the Drosophila cytochrome P450 Cyp6a2 enable DDT metabolism. Eur. J. Biochem..

[65] Kimura-Kuroda J., Komuta Y., Kuroda Y., Hayashi M., Kawano H. (2012). Nicotine-Like Effects of the neonicotinoid insecticides acetamiprid and imidacloprid on cerebellar neurons from neonatal rats. PLoS ONE.

[66] Cocco P., Blair A., Congia P., Saba G., Flore C., Ecca M.R., Palmas C. (1997). Proportional mortality of dichloro-diphenyl-trichloroethane (DDT) workers: A preliminary report. Arch. Environ. Health.

[67] Richardson J.R., Roy A., Shalat S.L., von Stein R.T., Hossain M.M., Buckley B., Gearing M., Levey A.I., German D.C. (2014). Elevated serum pesticide levels and risk for Alzheimer disease. JAMA Neurol..

